# Supplementary material for: The synergistic effect of Hf-O-Ru bonds and oxygen vacancies in Ru/HfO2 for enhanced hydrogen evolution
Source: Nat Commun. 2022 Mar 11;13:1270. doi: 10.1038/s41467-022-28947-9 (PMC8917135; doi:10.1038/s41467-022-28947-9)
Supplement: Supplementary file 1 — Supplementary Information [file 41467_2022_28947_MOESM1_ESM.pdf]

Supplementary Information

The Synergistic Effect of Hf-O-Ru Bonds and Oxygen Vacancies in Ru/HfO_2 for Enhanced Hydrogen Evolution

Guangkai Li[1,‡], Haeseong Jang[2,‡], Shangguo Liu[1,‡], Zijian Li[3], Min Gyu Kim[4], Qing Qin[1]*, Xien Liu[1]* & Jaephil Cho[2]*

[1]College of Chemical Engineering, Qingdao University of Science and Technology, Qingdao, China.

[2]Department of Energy Engineering, Department of Energy and Chemical Engineering, Ulsan National Institute of Science and Technology (UNIST), Ulsan, South Korea.

[3]Department of Chemistry, City University of Hong Kong, Hong Kong, China.

[4]Beamline Research Division, Pohang Accelerator Laboratory (PAL), Pohang, South Korea.

[‡]These authors contributed equally: Guangkai Li, Haeseong Jang, Shangguo Liu.

*email: qinqing@qust.edu.cn; liuxien@qust.edu.cn; jpcho@unist.ac.kr

Supplementary Methods

Chemicals

Ruthenium(III) chloride ($RuCl_3 \cdot xH_2O$), oleylamine ($C_{18}H_{37}N$), and polyvinyl pyrrolidone (PVP, MW = 58000) were purchased from Aladdin. Hafnium chloride ($HfCl_4$), and ethylene glycol ($C_2H_6O_2$) were purchased from macklin. KOH, ethylene glycol, ethanol and cyclohexane were obtained from Sinopharm Chemical Reagent Co. Ltd. All reagents were used as received without further purification.

Materials Characterization

The phases of the prepared catalysts were detected by the D/Max2000, Rigaku diffraction system with Cu Kα radiation. The morphologies were recorded using a field-emission scanning electron microscope with a model of Hitachi S4800. TEM and HRTEM images were recorded on a JEOL JEM-2100F high resolution transmission electron microscopy at 200 kV. The XPS tests were performed on an Escalab instrument (Escalab 250 xi, Thermo Scientific, England). XANES spectra and EXAFS spectra were measured using the BL10C beam line at the Pohang Light Source (PLS-II) in Korea.

Electrochemical measurements

The electrocatalytic hydrogen evolution reaction performance of the prepared catalysts and commercial Ru/C (Ru: 5 %), Pt/C (Pt: 20 %) were evaluated in a typical three-electrode electrochemical cell with a glassy carbon electrode (GCE) (diameter: 3 mm) as working electrode, a carbon rod as counter electrode and a Hg/HgO electrode as reference electrode. The 1.0 M KOH was used as the electrolyte. All potentials were

calibrated relatively to reversible hydrogen electrode (RHE) with 95% iR compensation. The calculation formula is $E_{RHE} = E_{Hg/HgO} + 0.059\ pH + 0.098$. All the polarization curves were recorded at a scan rate of 5 mV s^{-1} with an electrochemical window of -0.6 – 0 V (vs RHE). The EIS measurements were performed in a frequency ranged from 100 kHz – 0.1 Hz at the potential of -0.039 V (vs RHE). The durability tests were performed by a chronoamperometry method at a constant potential of -0.039 V (*vs.* RHE).

The catalyst ink was prepared according to the following steps. 2 mg of the catalyst, and 2 mg of XC 72 were dispersed in a mixed solvent of ethanol (0.2 mL) and ultrapure water (0.1 mL). Then, 40 μL of 5 wt % Nafion solution was added to the solution. After that, the mixture was ultrasonicated for 30 min to get a homogeneous ink. Finally, 5 μL of the ink was pipetted onto the GC disk to form a catalyst layer and dried naturally.

In situ and *operando* XAS measurement

In situ XANES and EXAFS experiments were performed at the BL10C beam line, the Pohang Light Source (PLS-II) in Korea. A home-made *operando* three electrode cell system, which consist of platinum counter electrode, Hg/HgO reference electrode, and electrocatalysts loaded working electrode, with polyimide film windows were employed. All *operando* XAS analysis was conducted under in-situ HER conditions in 1.0 M KOH electrolyte. To detect the structural stability, the *operando* XAS results were collected after conducting chronoamperometry (CA) test at -0.039 V vs RHE for 12 h.

Computational details

All density functional theory calculations in this study were performed by using the Vienna ab initio simulation package (VASP).[1] The Perdew-Burke-Ernzerhof (PBE)[2] functional was employed to treat the exchange-correlation interactions. The plane-wave basis set with a kinetic energy cutoff of 400 eV, the energy convergence criterion of 10^{-5} eV, the force convergence criterion of 0.02 eV $Å^{-1}$, and a (2×2×1) Monkhorst-Pack k-point sampling was employed for structure relaxation. Surface calculations were performed with Ru(001), HfO_2(001) and HfO_2(001) supported Ru clusters. For different surface models, the bottom layer was fixed at the converged bulk distance. A sufficiently large vacuum gap (> 10 Å) was employed to prevent the interaction between neighboring periodic structures along the c axis. H_2 and H_2O were calculated in boxes of 20 Å×20 Å×20 Å with the gamma point only. The CI-NEB method was adopted to search the minimum energy paths of H_2O dissociation reaction.[3,4] Six images were used for CI-NEB calculations. The free energy diagrams for HER were calculated with reference to the computational hydrogen electrode.[5] The free energy of gas phase and adsorbed species can be obtained from the following equation:

$$G = E_{\mathrm{elec}} + ZPE - TS \quad (1)$$

where E_{elec} is the electronic energy.

The d band center (ε) was calculated based on the following equation:

$$\varepsilon = \frac{\int_{-\infty}^{\infty} \rho(x) x dx}{\int_{-\infty}^{\infty} \rho(x) dx} \quad (2)$$

where ρ(x) is the PDOS at the energy of x.

Supplementary Figures

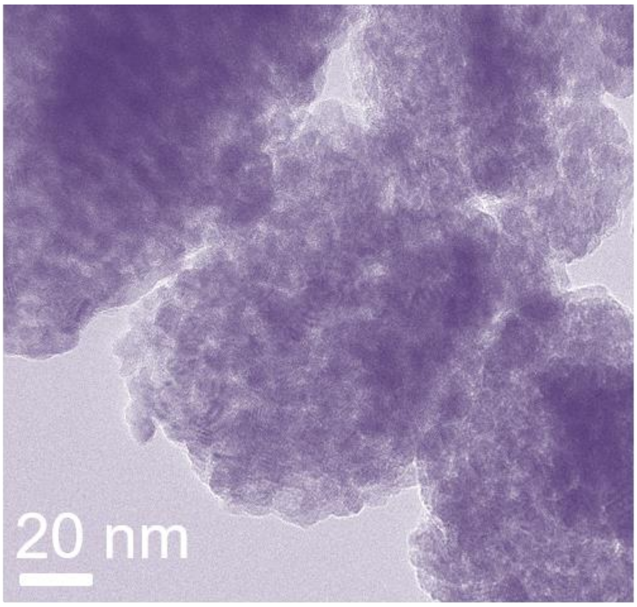

Supplementary Fig. 1 Morphology characterization. The TEM image of the V_O-Ru/HfO_2-OP catalyst.

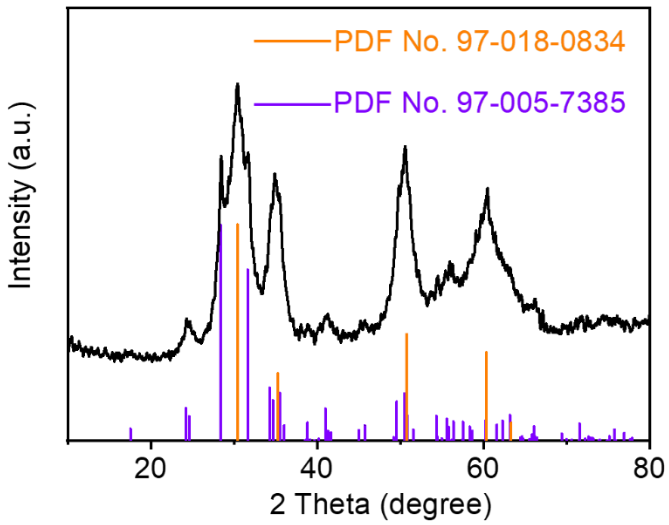

Supplementary Fig. 2 Phase characterization. XRD pattern of the prepared pristine HfO_2.

The XRD pattern of pristine HfO_2 was perfectly indexed to a mixture of Monoclinic (PDF No. 97-005-7385) and Cubic (PDF No. 97-018-0834) phase HfO_2.

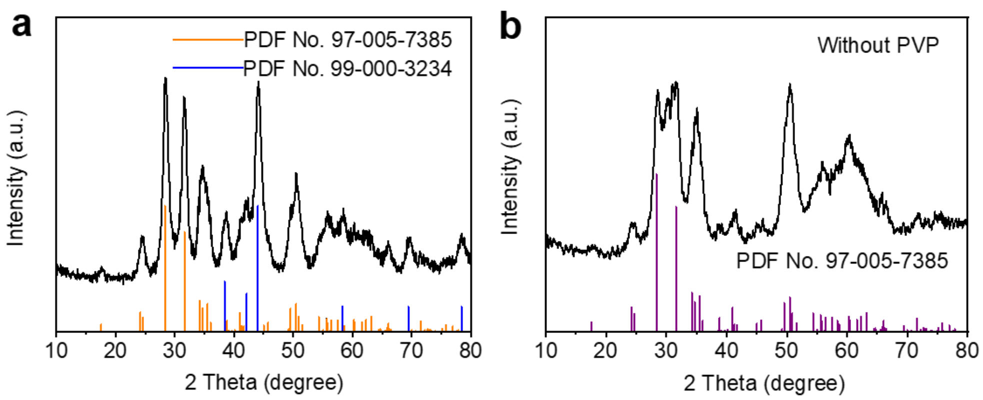

Supplementary Fig. 3 Phase characterizations. a The XRD pattern of V_O-Ru/HfO_2-P, **b** The XRD pattern of V_O-Ru/HfO_2-O.

As shown in Supplementary Fig. 3a, the XRD pattern of catalyst V_O-Ru/HfO_2-P prepared with PVP shows obvious diffraction peaks corresponding to hexagonal crystal structure of Ru (PDF No. 99-000-3234) and Monoclinic HfO_2 (PDF No. 97-005-7385). The average grain size of Ru in Ru/HfO_2-P is 7 nm, corroborated by Supplementary Figs. 4-5.

The XRD pattern of V_O-Ru/HfO_2-O prepared with oleylamine shows the characteristic peaks can be well indexed to Monoclinic HfO_2 (PDF No. 97-005-7385), but no diffraction peaks assigned to Ru can be observed, indicating the well dispersion and stabilization of ultra-small Ru nanoclusters by oleylamine during the synthetic process. However, the agglomeration of HfO_2 appears in the V_O-Ru/HfO_2-O samples (Supplementary Fig. 6), implying the key role of PVP played in stabilizing the HfO_2 nanoparticles.

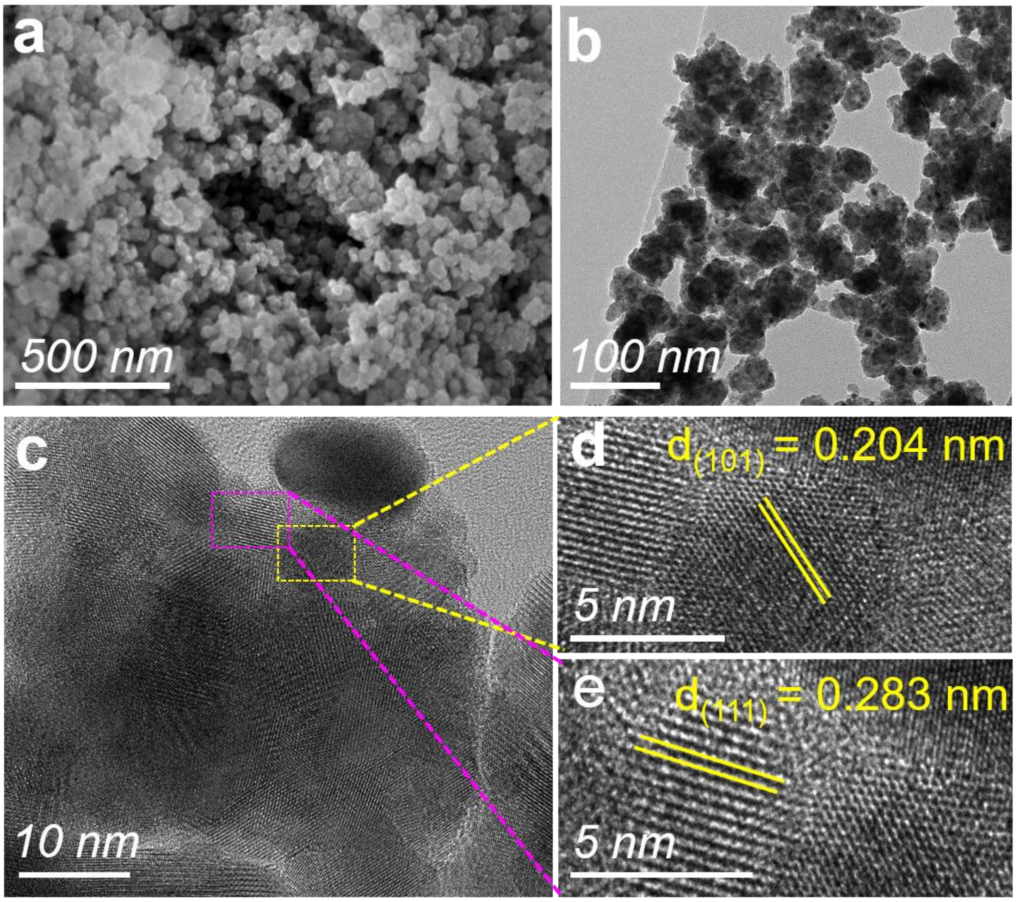

Supplementary Fig. 4 Morphology and structural characterizations of Vo-Ru/HfO$_2$-P. a SEM image, **b** TEM image, **c** HRTEM image, **d** and **e** Magnified HRTEM images corresponding to the regions in supplementary Fig. 4c.

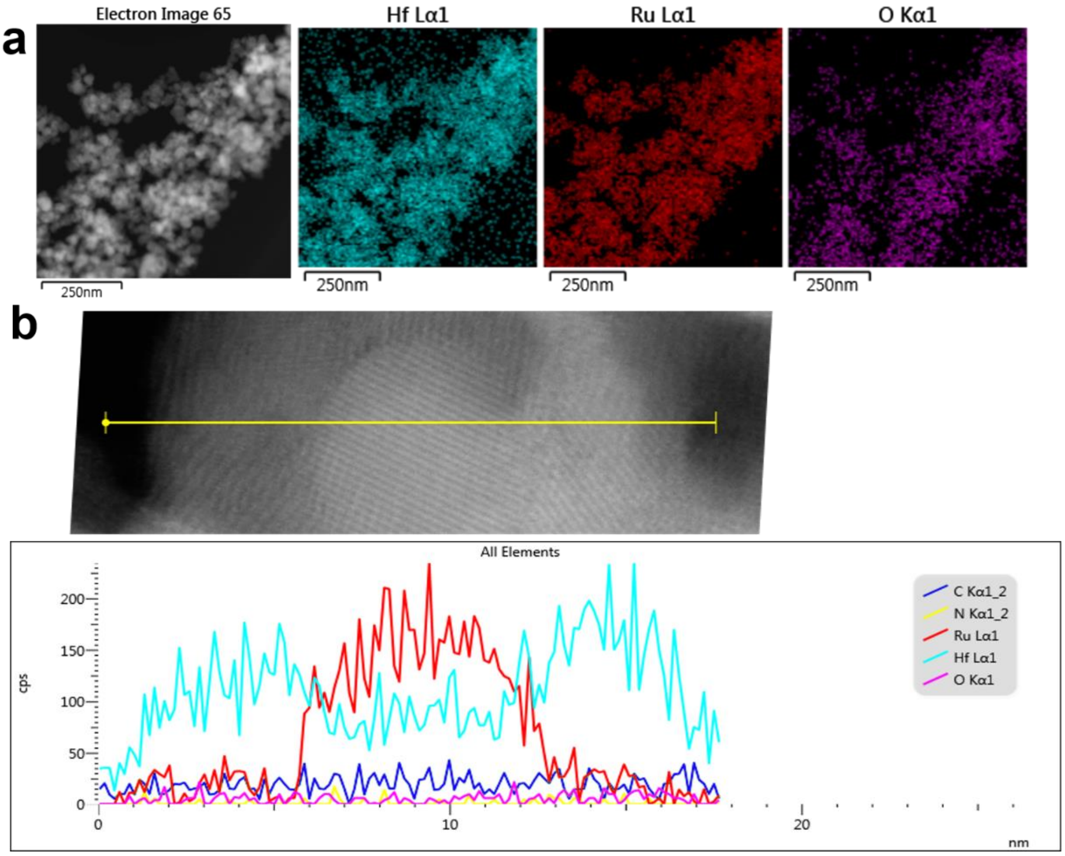

Supplementary Fig. 5 Elemental composition of V_O-Ru/HfO_2-P. a EDS elemental mappings, **b** EDS elemental linear scanning.

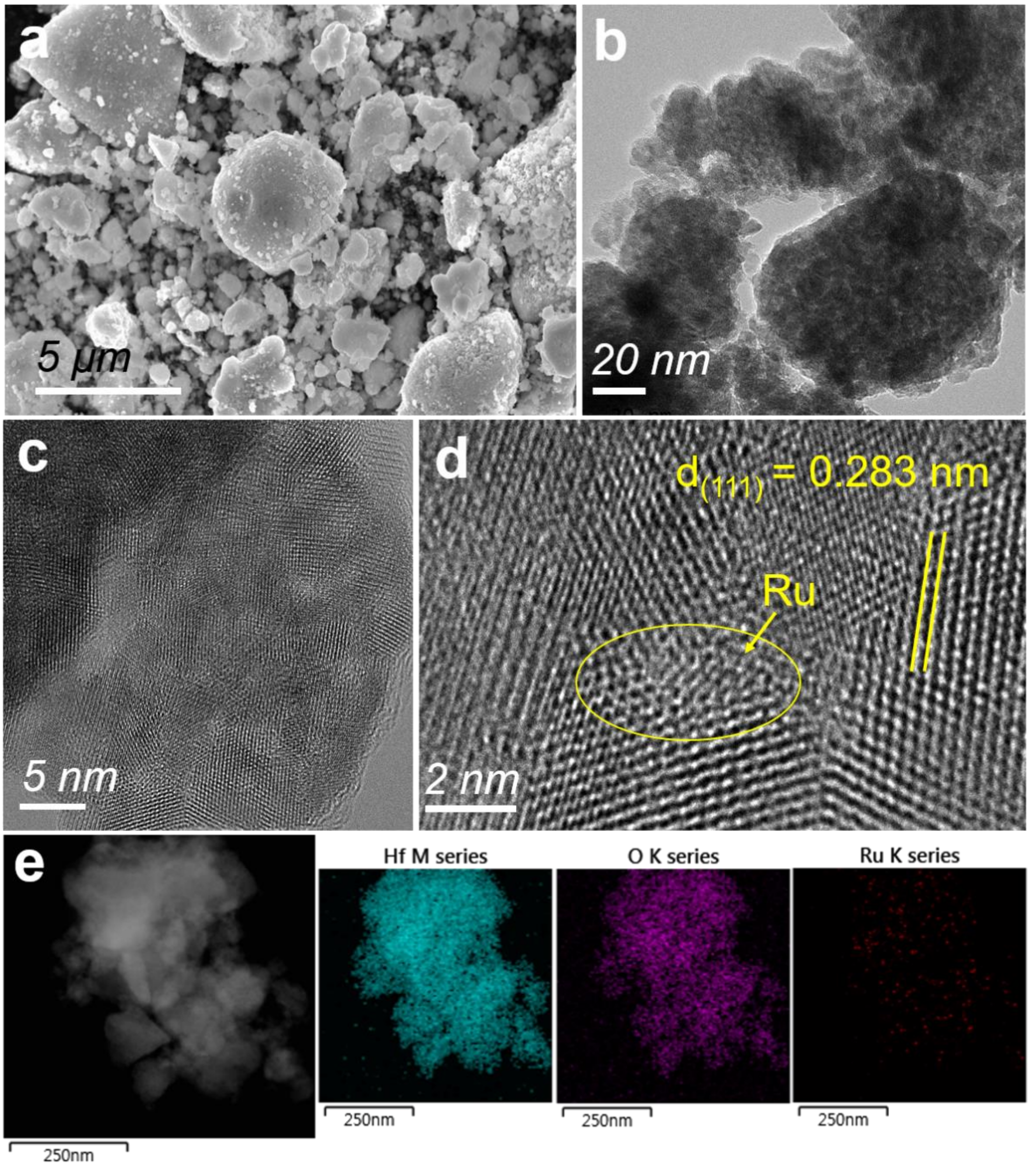

Supplementary Fig. 6 Morphology and structural characterizations of V_O-Ru/HfO_2-O. a SEM image, **b** TEM image, **c** HRTEM image, **d** Magnified HRTEM image, **e** Elemental mappings.

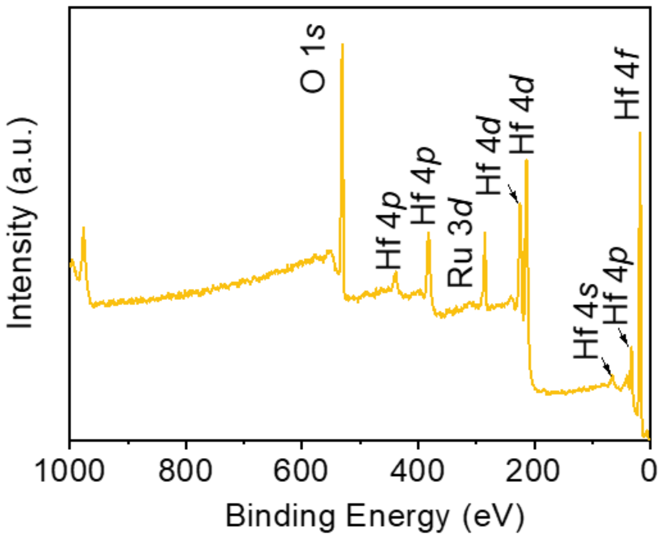

Supplementary Fig. 7 Surface elemental composition analysis. XPS survey spectrum of V_O-Ru/HfO_2-OP.

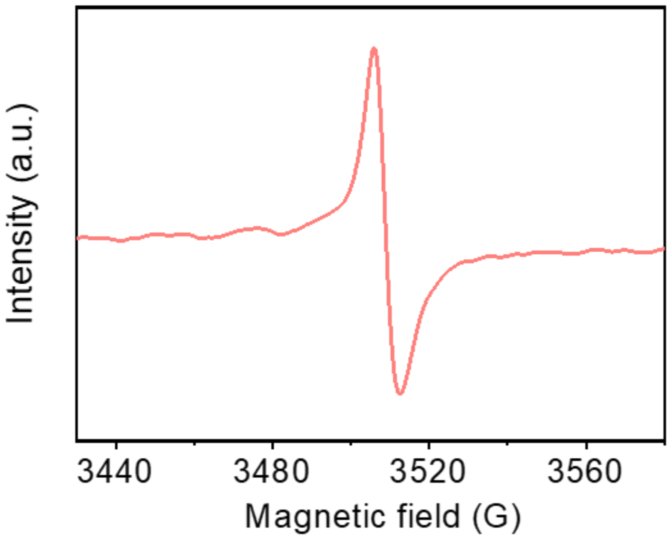

Supplementary Fig. 8 Oxygen vacancy characterization. EPR spectrum of the V_O-Ru/HfO_2-OP.

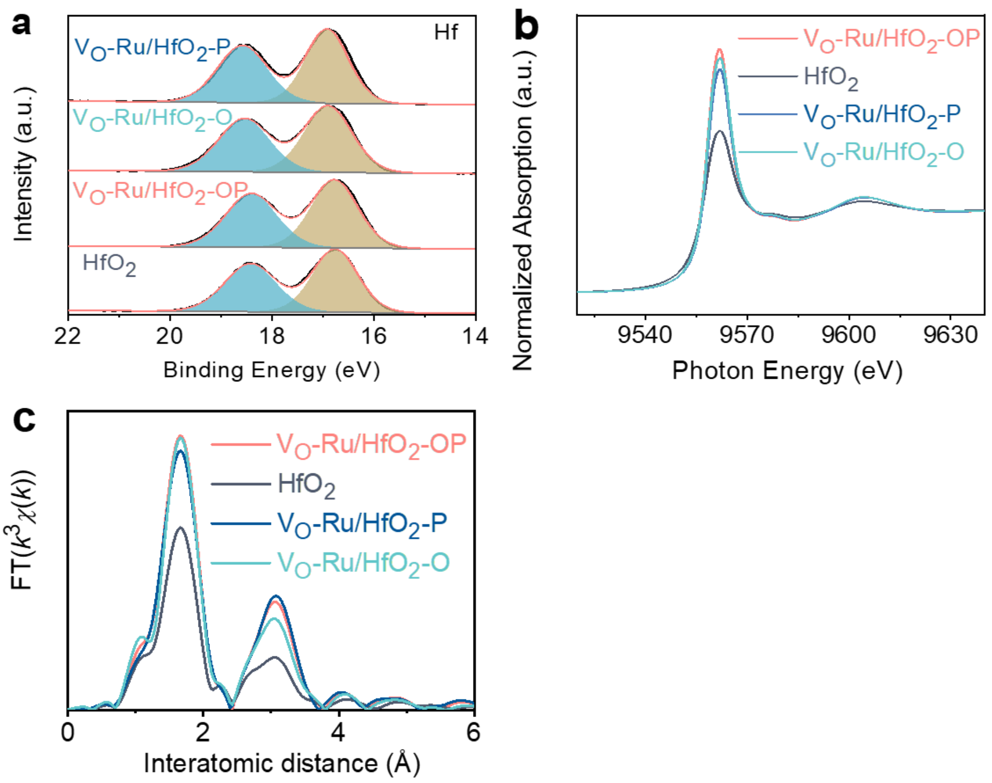

Supplementary Fig. 9 Electronic and fine structural characterizations. a High resolution XPS spectra of Hf 4*f*. **b** XANES spectra for Hf L_3-edge of HfO_2, V_O-Ru/HfO_2-OP, V_O-Ru/HfO_2-O, and V_O-Ru/HfO_2-P. **c** EXAFS spectra for Hf L_3-edge of HfO_2, V_O-Ru/HfO_2-OP, V_O-Ru/HfO_2-O, and V_O-Ru/HfO_2-P.

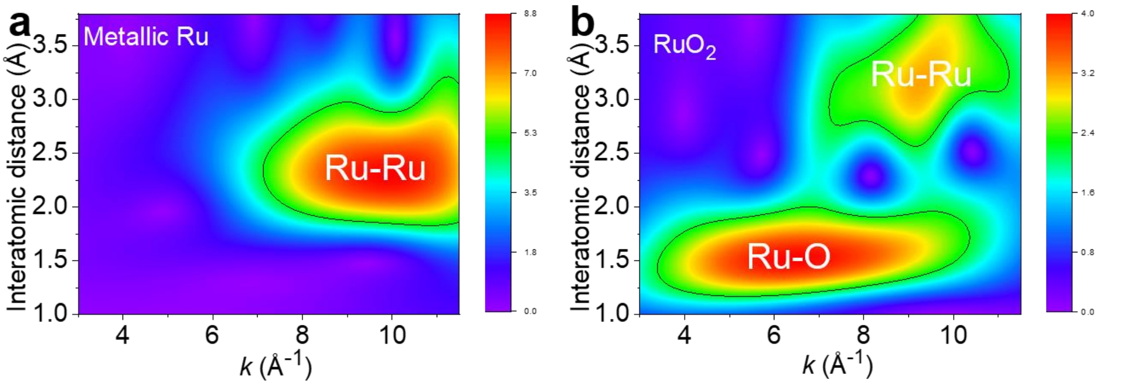

Supplementary Fig. 10 Wavelet transform of references. a metallic Ru, and **b** RuO_2.

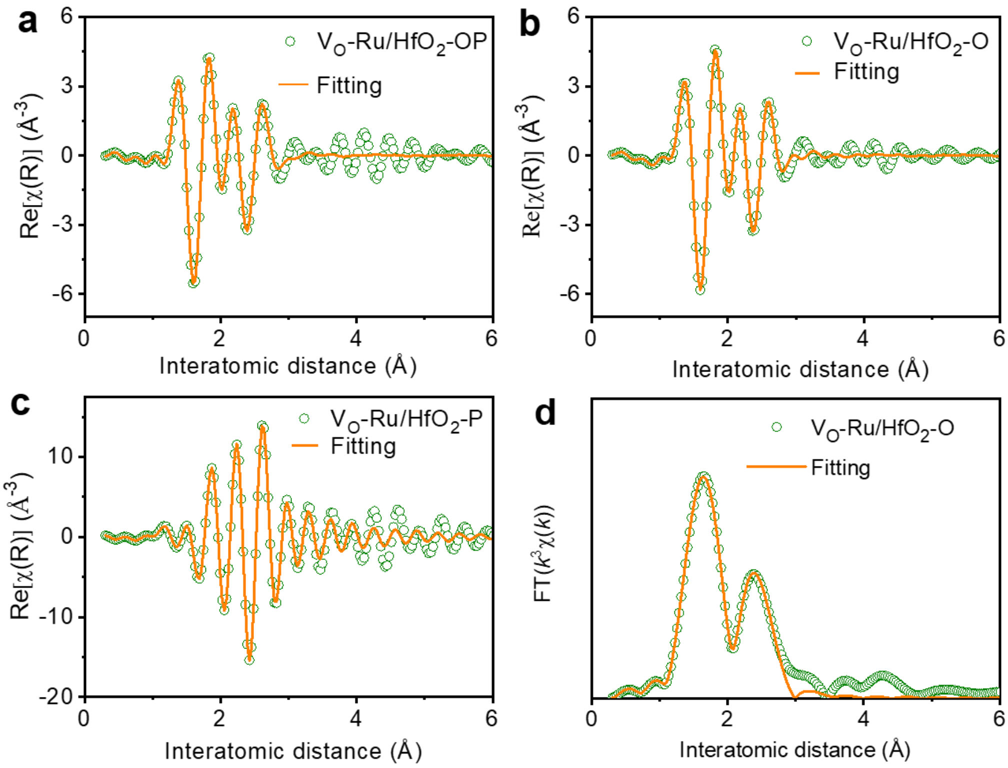

Supplementary Fig. 11 EXAFS curves and corresponding fitting results of prepared catalysts. **a** The corresponding experimental curve and fitting result of EXAFS k space of V_O-Ru/HfO_2-OP. **b** The corresponding experimental curve and fitting result of EXAFS k space of V_O-Ru/HfO_2-O. **c** The corresponding experimental curve and fitting result of EXAFS k space of V_O-Ru/HfO_2-P. **d** The corresponding experimental curve and fitting result of EXAFS R space of V_O-Ru/HfO_2-O.

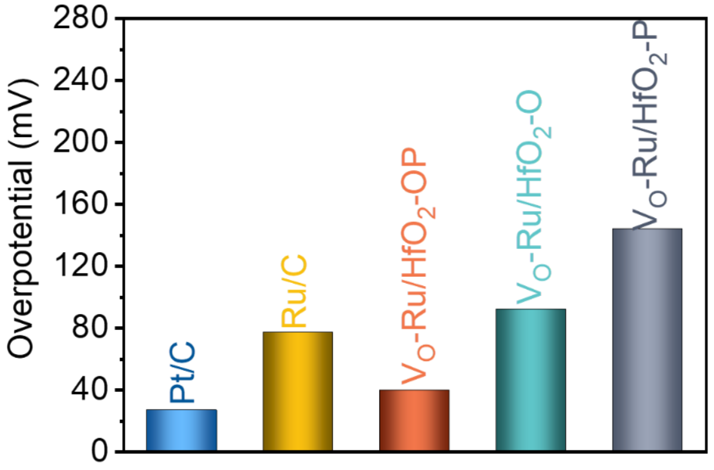

Supplementary Fig. 12 The electrocatalytic HER activities. Comparison of catalytic activities for V_O-Ru/HfO_2-OP, V_O-Ru/HfO_2-O, V_O-Ru/HfO_2-P, Ru/C, and Pt/C using η_{10} as the indicator.

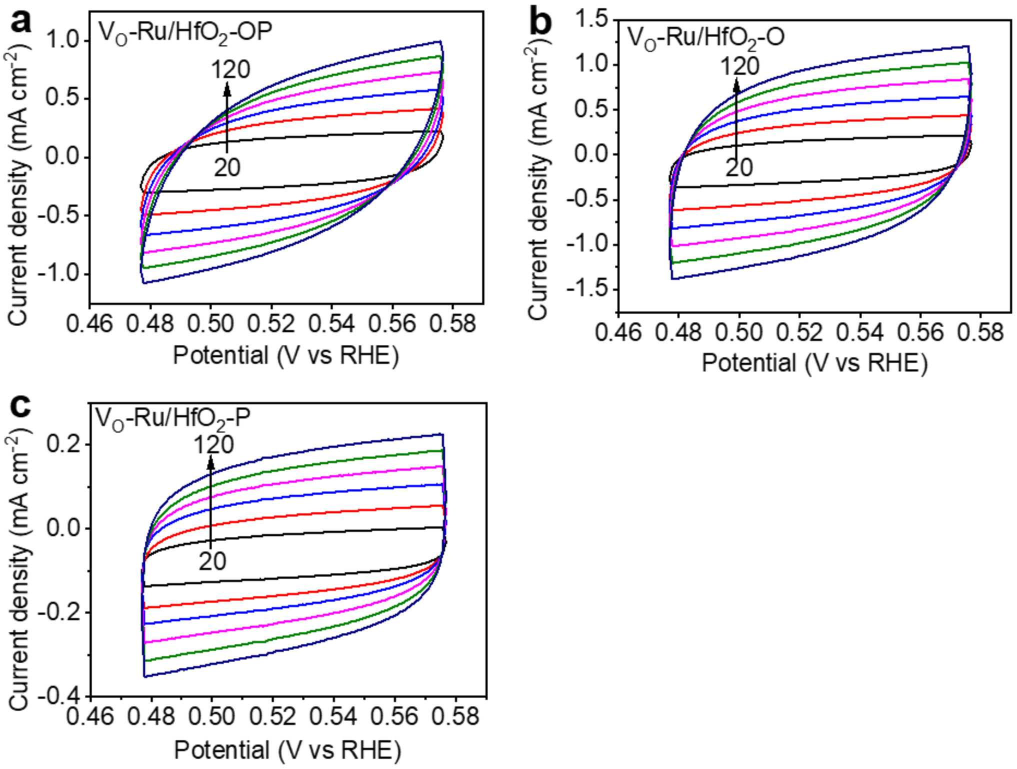

Supplementary Fig. 13 Cyclic voltammograms at various scan rates. a V_O-Ru/HfO_2-OP, **b** V_O-Ru/HfO_2-O, **c** V_O-Ru/HfO_2-P electrodes

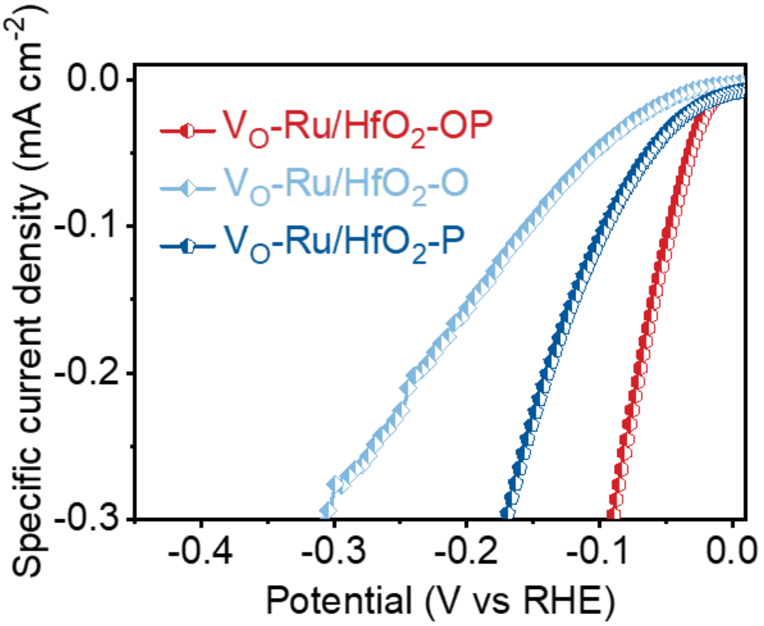

Supplementary Fig. 14 The HER activity normalized to the ECSA surface areas.

HER polarization curves of V_O-Ru/HfO_2-OP, V_O-Ru/HfO_2-O, and V_O-Ru/HfO_2-P.

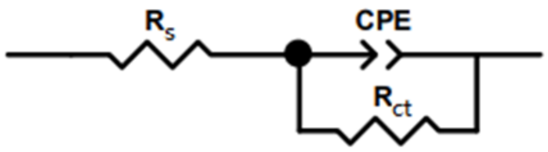

Supplementary Fig. 15 Equivalent circuit was used for simulating the Nyquist plots in Fig. 3e. R_s, R_{ct} and CPE represent the solution resistance, the charge transfer resistance and constant phase element, respectively.

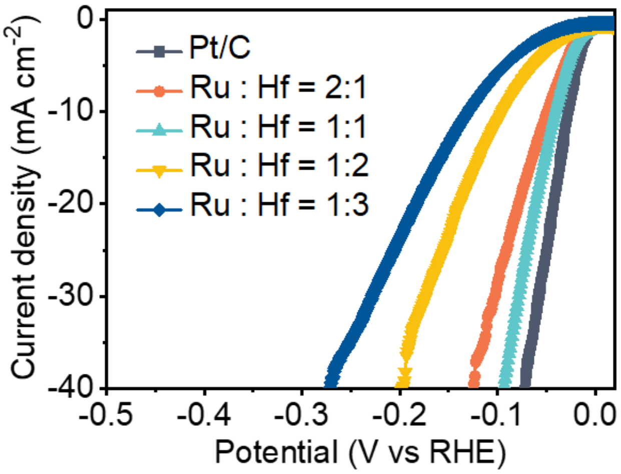

Supplementary Fig. 16 Study for the optimal molar ratio of raw material. The polarization curves of the catalysts prepared with different molar ratio of Ru to Hf.

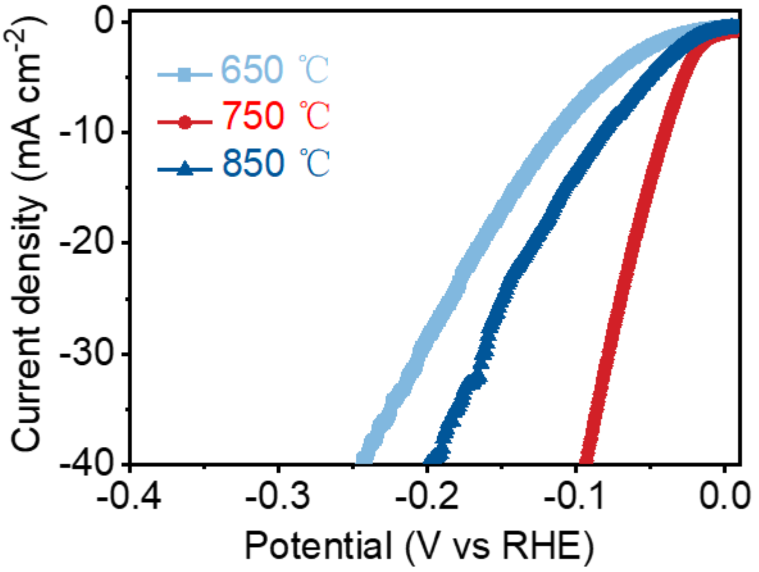

Supplementary Fig. 17 Study for the optimal annealing temperature. The polarization curves of V_O-Ru/HfO_2-OP prepared at different annealing temperature.

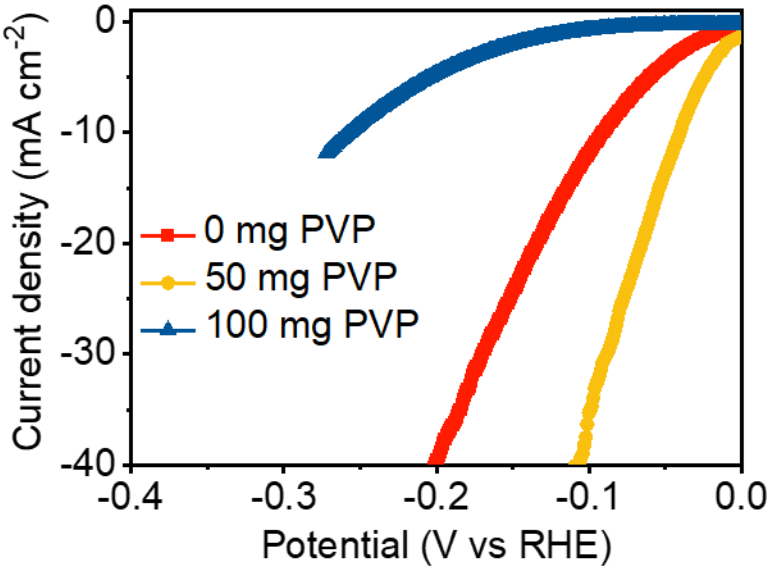

Supplementary Fig. 18 Study for the optimal PVP dosage. The polarization curves of the catalysts prepared with different amount of PVP.

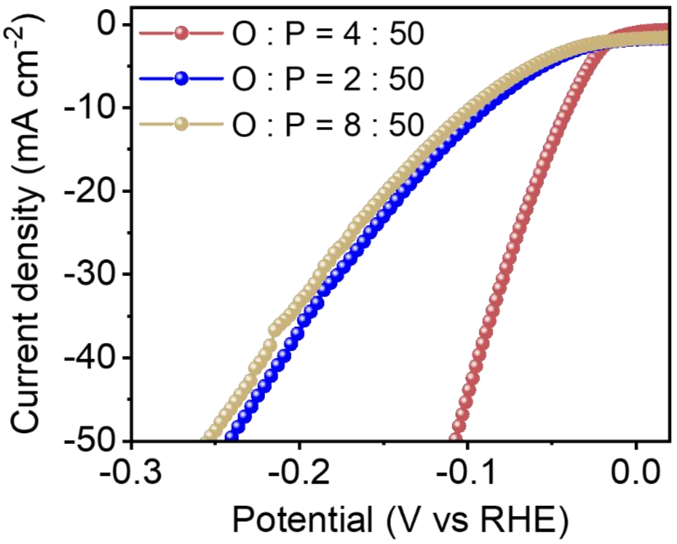

Supplementary Fig. 19 Study for the optimal ratio of O (oleylamine) to P (PVP). The polarization curves of the catalysts prepared with different ratio of O (oleylamine) to P (PVP).

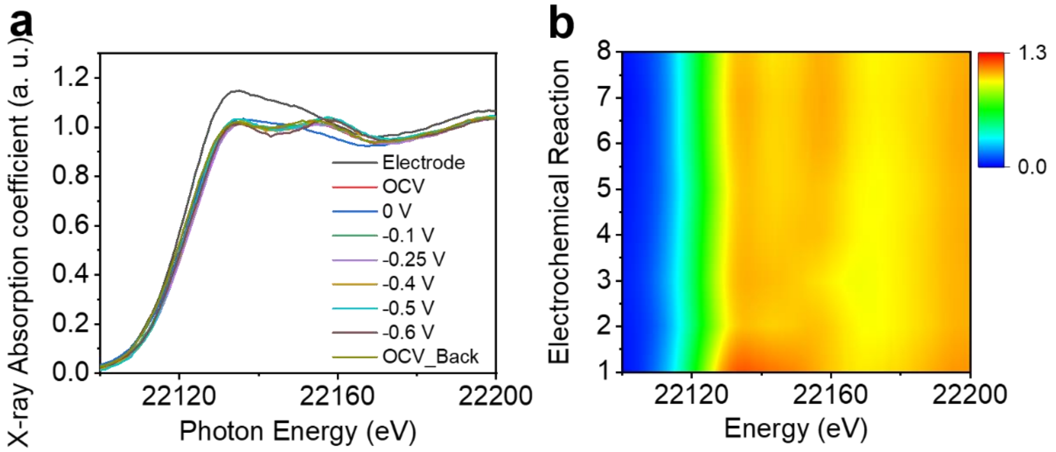

Supplementary Fig. 20 *Operando* Ru K-edge XANES characterization. a *Operando* Ru K-edge XANES spectra of V_O-Ru/HfO_2-OP, and **b** the corresponding two dimensional (2D) color map.

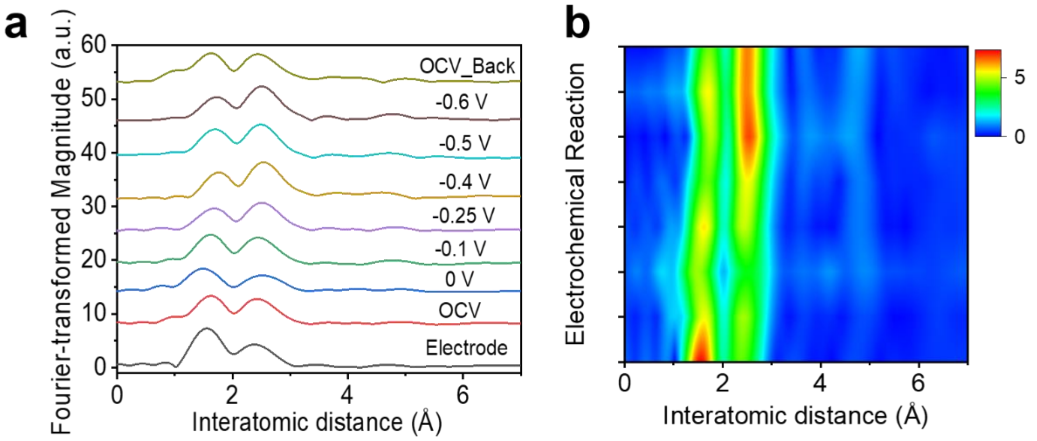

Supplementary Fig. 21 ***Operando*** **Ru K-edge FT-EXAFS characterization. a** *Operando* Ru K-edge FT-EXAFS spectra of V_O-Ru/HfO_2-OP, and **b** the corresponding two dimensional (2D) color map.

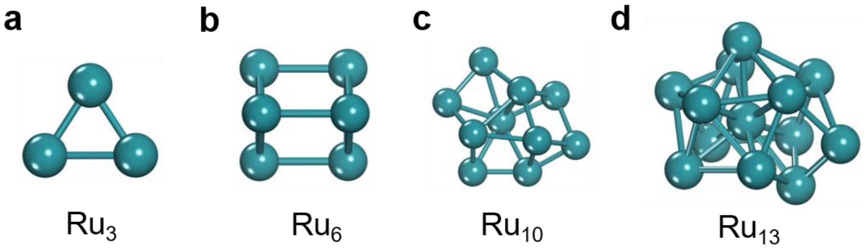

Supplementary Fig. 22 The DFT Calculated structural model for Ru clusters. a Ru_3, **b** Ru_6, **c** Ru_{10} and **d** Ru_{13} clusters.

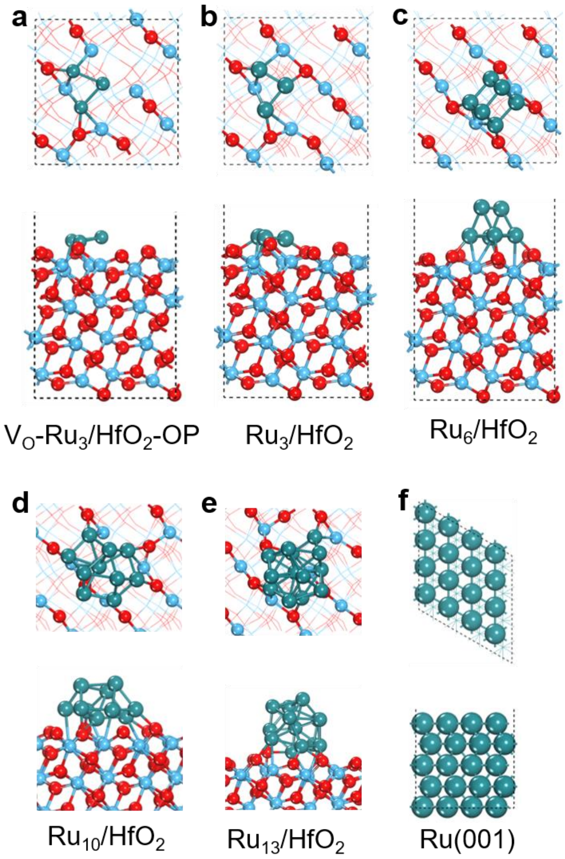

Supplementary Fig. 23 The DFT Calculated structural model for supported Ru clusters and Ru(001). Top and side view of the relaxed atomic models of **a** V_O-Ru_3/HfO_2-OP, **b** Ru_3/HfO_2, **c** Ru_6/HfO_2, **d** Ru_{10}/HfO_2, **e** Ru_{13}/HfO_2 and **f** Ru(001).

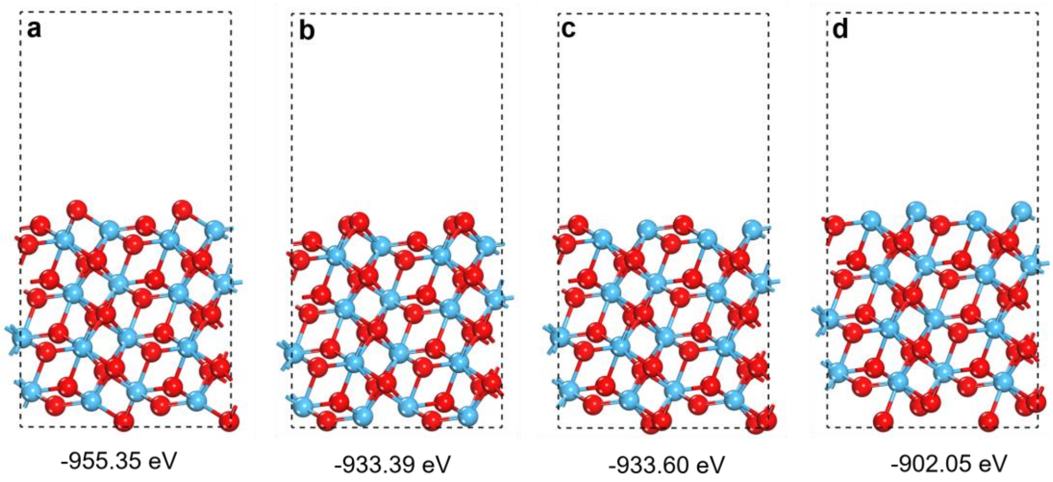

Supplementary Fig. 24 The total energy of HfO_2(001) with different atomic Terminal. **a**, **b** O terminated HfO_2(001) surface and **c**, **d** Hf terminated HfO_2(001) surface.

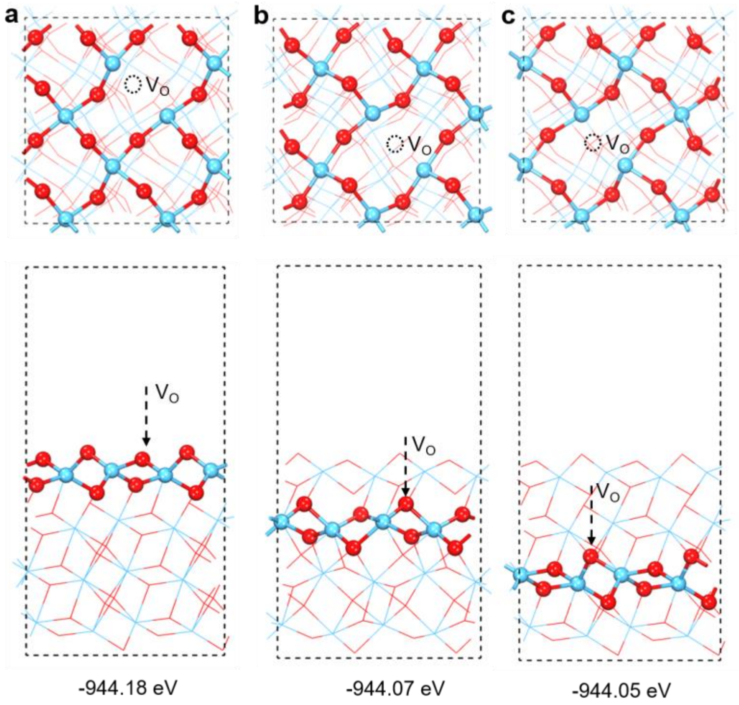

Supplementary Fig. 25 The total energy of V_O-HfO_2(001) with different V_O localized place. **a** The V_O localised on the first layer of HfO_2(001), **b** The V_O localised on the second layer of HfO_2(001), and **c** The V_O localised on the third layer of HfO_2(001).

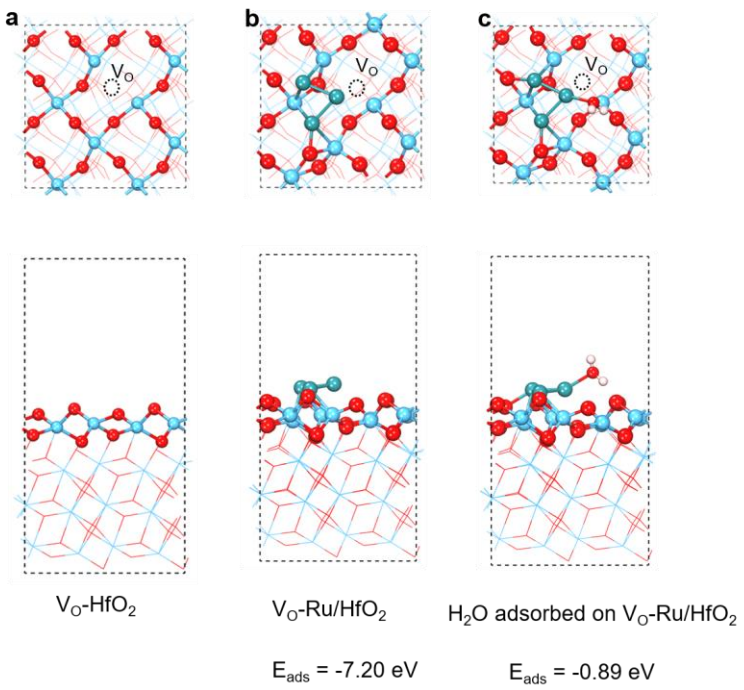

Supplementary Fig. 26 The DFT Calculated structural model for V_O-HfO_2(001) and calculated adsorbed energy. **a** Structural representations of V_O-HfO_2, **b** Ru cluster adsorbed on V_O-HfO_2 and **c** H_2O adsorbed on V_O-Ru/HfO_2.

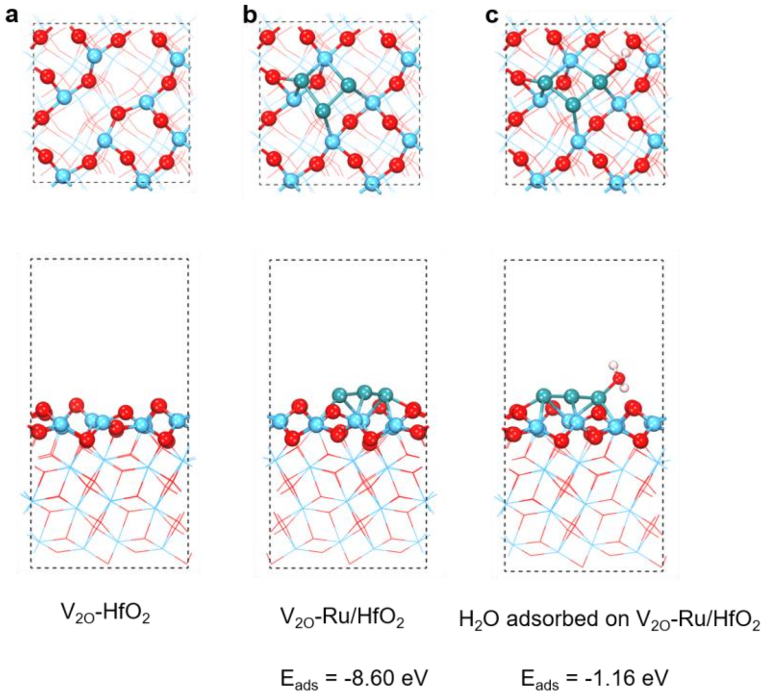

Supplementary Fig. 27 The DFT Calculated structural model for V_{2O}-HfO_2(001) and calculated adsorbed energy. **a** Structural representations of V_{2O}-HfO_2, **b** Ru cluster adsorbed on V_{2O}-HfO_2 and **c** H_2O adsorbed on V_{2O}-Ru/HfO_2.

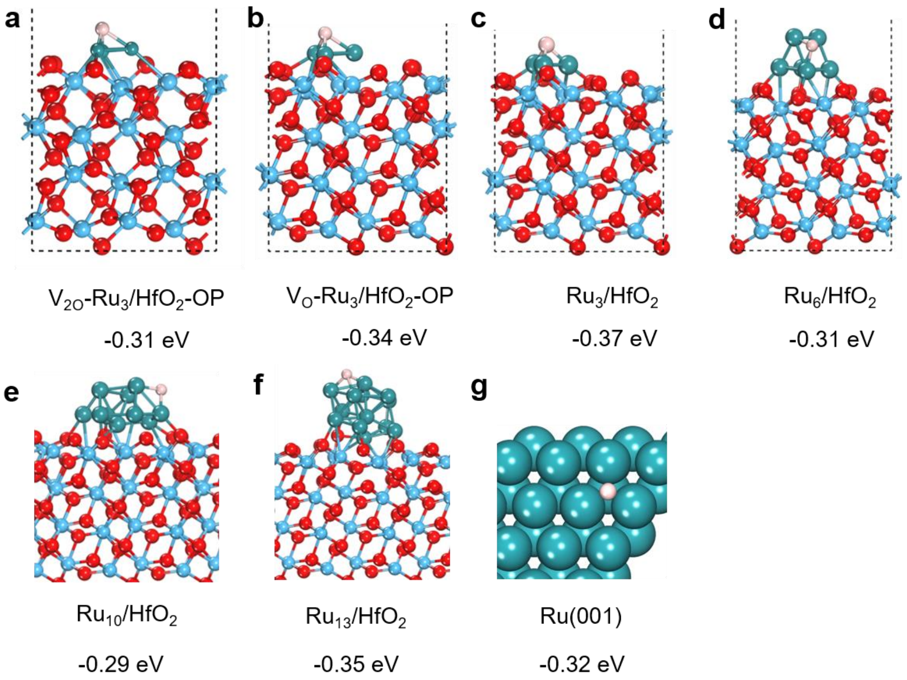

Supplementary Fig. 28 The calculated adsorption free energy of H* on different catalysts. **a** V_{2O}-Ru_3/HfO_2-OP, **b** V_O-Ru_3/HfO_2-OP, **c** Ru_3/HfO_2, **d** Ru_6/HfO_2, **e** Ru_{10}/HfO_2, **f** Ru_{13}/HfO_2 and **g** Ru(001).

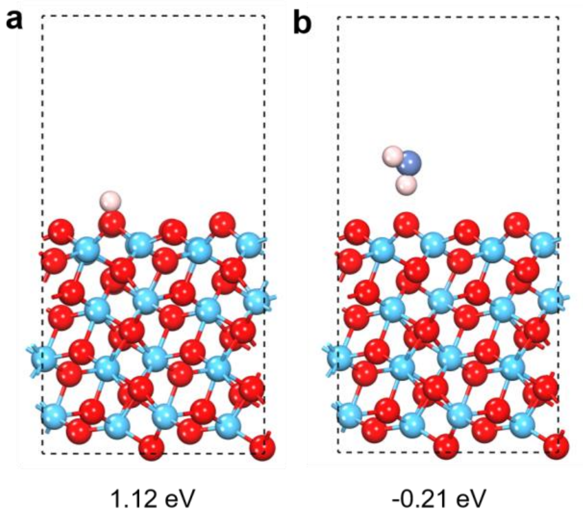

Supplementary Fig. 29 The calculated adsorption energy of H* and H_2O on HfO_2(001). **a** The adsorption free energy of H*, **b** the adsorption energy of H_2O.

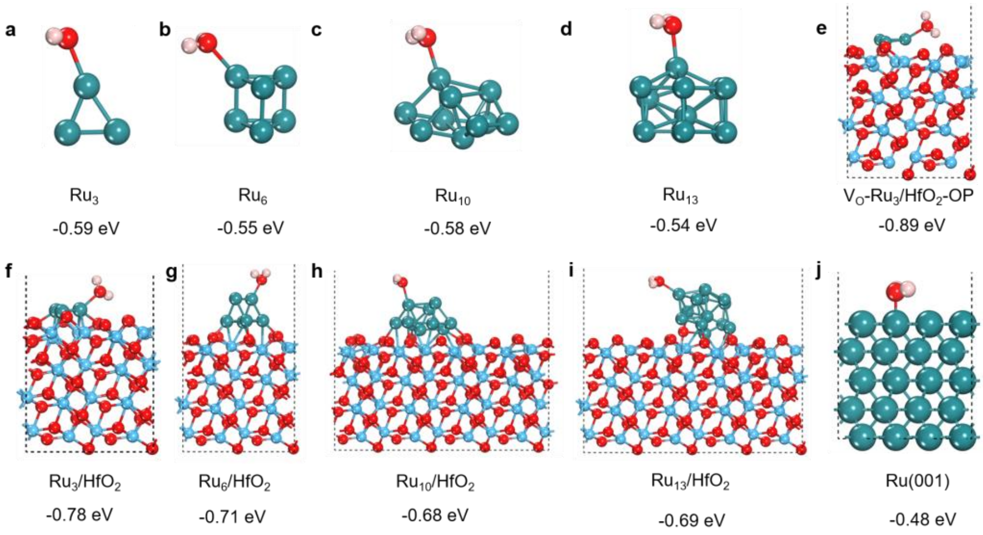

Supplementary Fig. 30 The DFT calculated adsorption energy of H_2O on Ru-based catalysts. a Ru_3, **b** Ru_6, **c** Ru_{10}, **d** Ru_{13}, **e** V_O-Ru_3/HfO_2-OP, **f** Ru_3/HfO_2**,** **g** Ru_6/HfO_2, **h** Ru_{10}/HfO_2, **i** Ru_{13}/HfO_2, and **j** Ru(001).

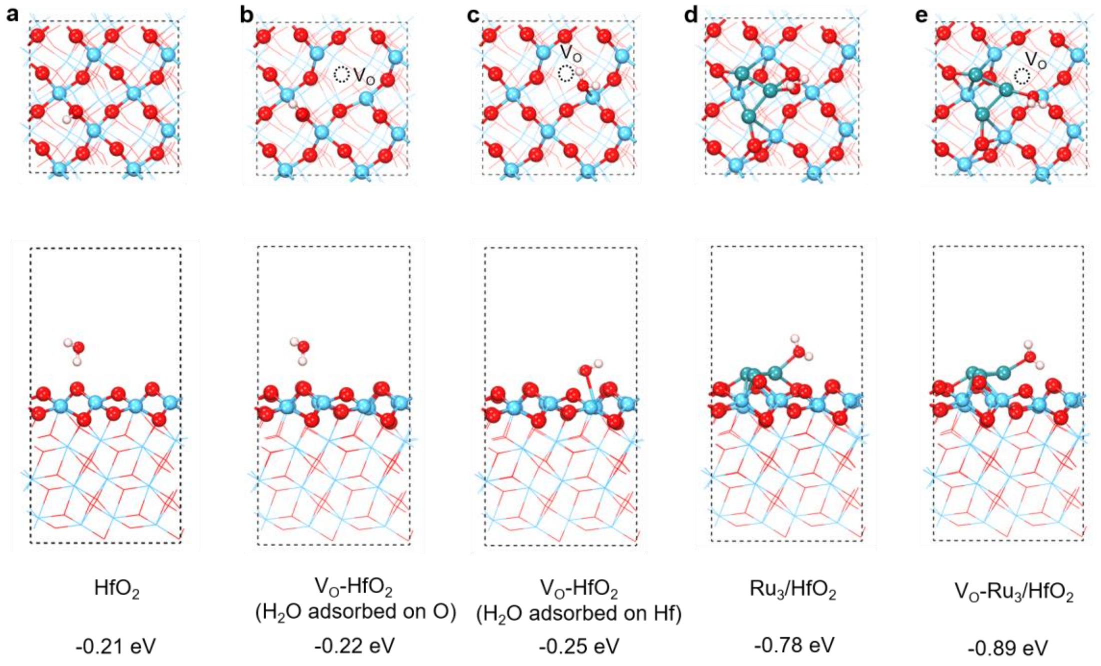

Supplementary Fig. 31 The calculated adsorption energy of H_2O on different catalysts. a HfO_2, **b** V_O-HfO_2 (O), **c** V_O-HfO_2 (Hf), **d** Ru_3/HfO_2, **e** V_O-Ru_3/HfO_2.

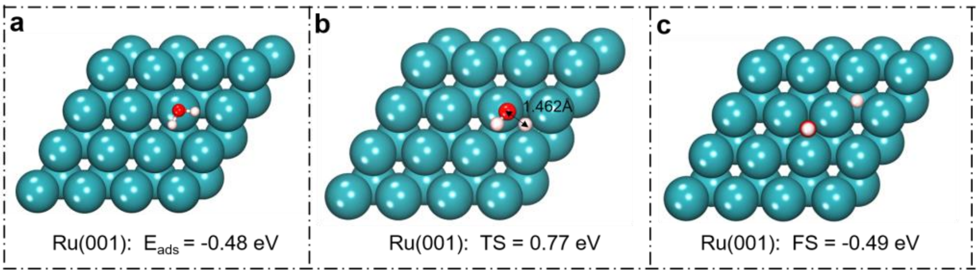

Supplementary Fig. 32 The energy barrier for water dissociation on Ru(001). a initial state, **b** transition state, **c** final state.

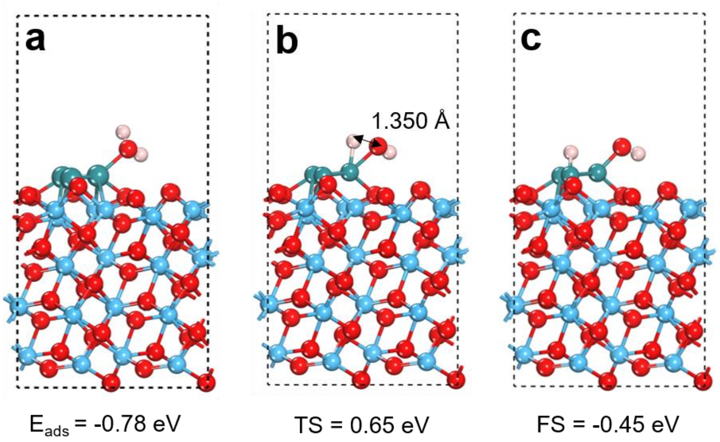

Supplementary Fig. 33 The energy barrier for water dissociation on $Ru_3@HfO_2$. a initial state, **b** transition state, and **c** final state.

.

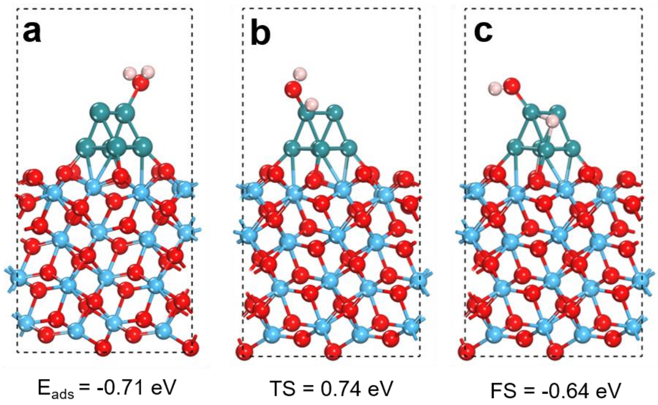

Supplementary Fig. 34 The energy barrier for water dissociation on Ru_6@HfO_2. a initial state, **b** transition state, and **c** final state.

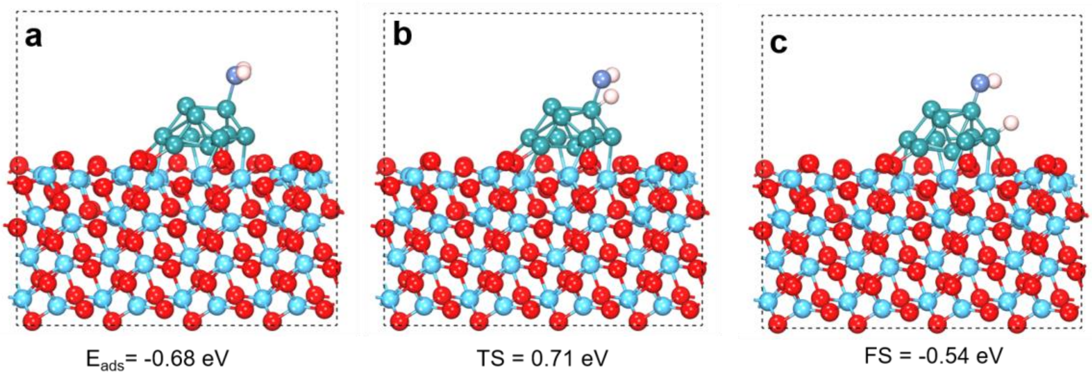

Supplementary Fig. 35 The energy barrier for water dissociation on Ru_{10}@HfO_2.

a initial state, **b** transition state, and **c** final state.

.

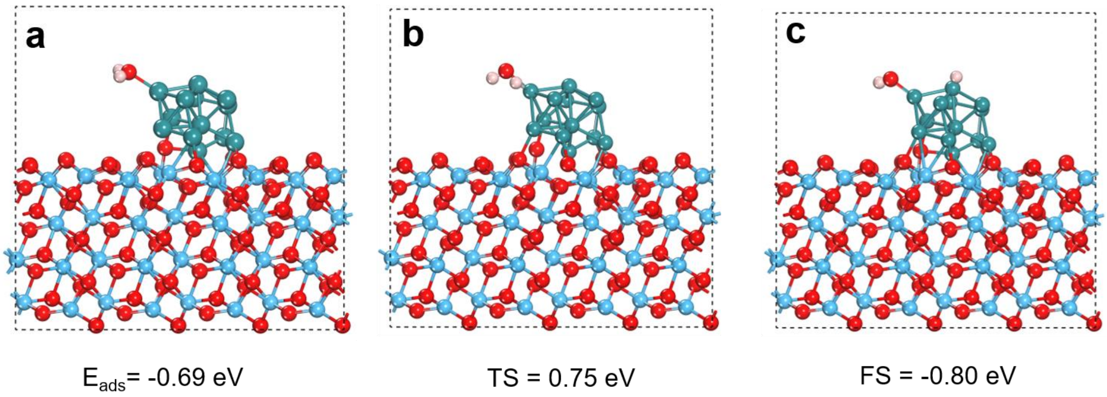

Supplementary Fig. 36 The energy barrier for water dissociation on Ru_{13}@HfO_2.

a initial state, **b** transition state, and **c** final state.

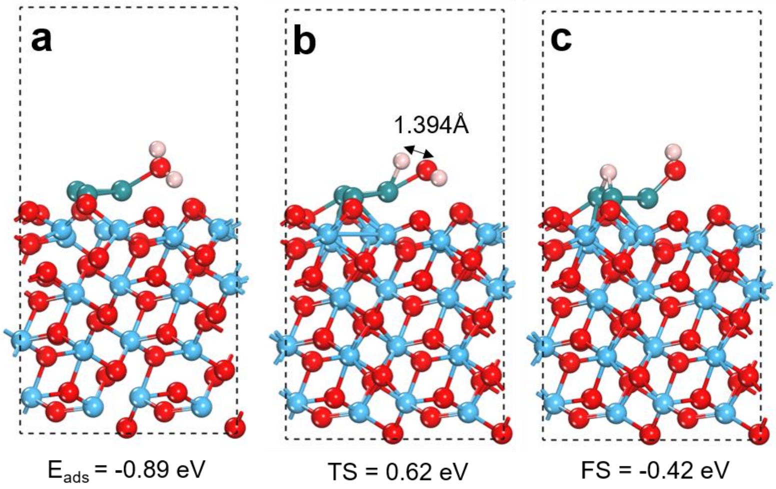

Supplementary Fig. 37 The energy barrier for water dissociation on V_O-Ru/HfO_2-OP. **a** initial state, **b** transition state, and **c** final state.

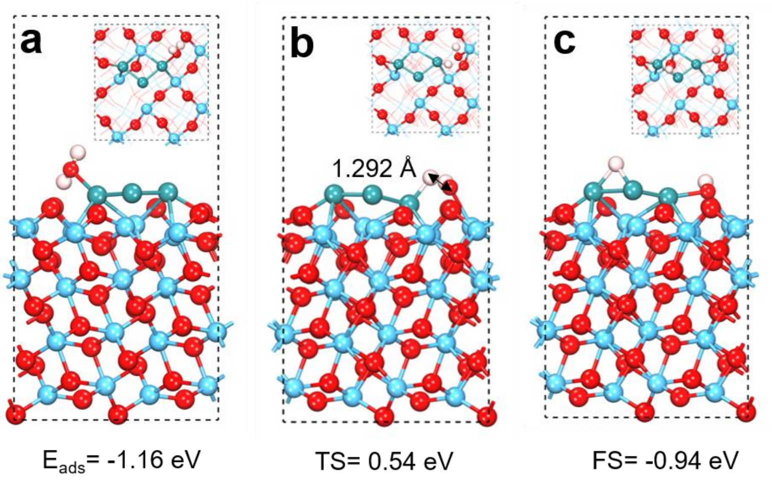

Supplementary Fig. 38 The energy barrier for water dissociation on V_{2O}-Ru/HfO_2-OP. a initial state, **b** transition state, and **c** final state.

Supplementary Tables

Supplementary Table 1 Structure parameters extracted from the Ru K-edge EXAFS curves Fitting.

Catalysts	Bond	Coordination number (CN)	Bond length R (Å)	σ^2 (Å) x 10^{-3}	E_0 (eV)	R factor
**Ru foil**	Ru-M	12	2.662	4.0	2.5	0.00169
**RuO_2**	Ru-O	6	1.980	1.5	1.1	0.00099
**V_O-Ru/HfO_2-O**	Ru-M	5.0	2.645	8.8	4.9	0.00343
	Ru-O	2.9	2.023	1.5	2.8	0.00066
**V_O-Ru/HfO_2-OP**	Ru-M	4.9	2.653	8.3	4.2	0.00379
	Ru-O	3.4	2.022	1.0	2.9	0.00116
**V_O-Ru/HfO_2-P**	Ru-M	11.0	2.674	4.5	6.9	0.00376
	Ru-O	0.48	2.352	7.1	6.1	0.00076

Oxygen = O, Ru or Hf = M

Supplementary Table 2 Electrochemical impedance parameters obtained simulating the Nyquist plots to the equivalent circuit model in Supplementary Fig. 15

Catalyst	R_S (ohm)	CPE-T (F)	CPE-P (F)	R_{ct} (ohm)
V_O-Ru/HfO_2-OP	9.096	0.001202	0.73969	49.1
Ru/C	9.34	0.0056874	0.47536	116.5
V_O-Ru/HfO_2-O	9.765	0.0021089	0.70255	200.1
V_O-Ru/HfO_2-P	8.017	0.0012319	0.64013	301.7
HfO_2	10.72	0.00077802	0.79177	4276.0

Supplementary Table 3 Summary for the adsorption energy of Ru cluster adsorbed on HfO_2, V_O-HfO_2 and V_{2O}-HfO_2 (E_{ads_Ru}); The adsorption energy of H_2O adsorbed on Ru/HfO_2, V_O-Ru_3/HfO_2-OP and V_{2O}-Ru_3/HfO_2-OP (E_{ads_H2O}); and the Kinetic barrier of water dissociation on the active sites of Ru/HfO_2, V_O-Ru_3/HfO_2-OP and V_{2O}-Ru_3/HfO_2-OP (E_{TS}).

	**Ru/HfO_2**	**V_O-Ru_3/HfO_2-OP**	**V_{2O}-Ru_3/HfO_2-OP**
**E_{ads_Ru}**	-5.63 eV	-7.20 eV	-8.60 eV
**E_{ads_H2O}**	-0.78	-0.89	-1.16 eV
**E_{TS}**	0.65	0.62	0.54

Supplementary References

1. Kresse, G. et al. Efficient iterative schemes for ab initio total-energy calculations using a plane-wave basis set. *Phys. Rev. B* **54**, 11169 (1996).
2. Perdew, J. P. et al. Generalized gradient approximation made simple. *Phys. Rev. Lett.* **77**, 3865 (1996).
3. Henkelman, G. et al. Improved tangent estimate in the nudged elastic band method for finding minimum energy paths and saddle points. *J. Chem. Phys.* **113**, 9978-9985 (2000).
4. Henkelman, G. et al. A climbing image nudged elastic band method for finding saddle points and minimum energy paths. *J. Chem. Phys.* **113**, 9901-9904 (2000).
5. Nørskov, J. K. et al. Origin of the overpotential for oxygen reduction at a fuel-cell cathode, *J. Phys. Chem. B* **108**, 17886–17892 (2004).
