# Supplementary figures and images for: The synergistic effect of Hf-O-Ru bonds and oxygen vacancies in Ru/HfO2 for enhanced hydrogen evolution
Source: Nat Commun. 2022 Mar 11;13:1270. doi: 10.1038/s41467-022-28947-9 (PMC8917135; doi:10.1038/s41467-022-28947-9)

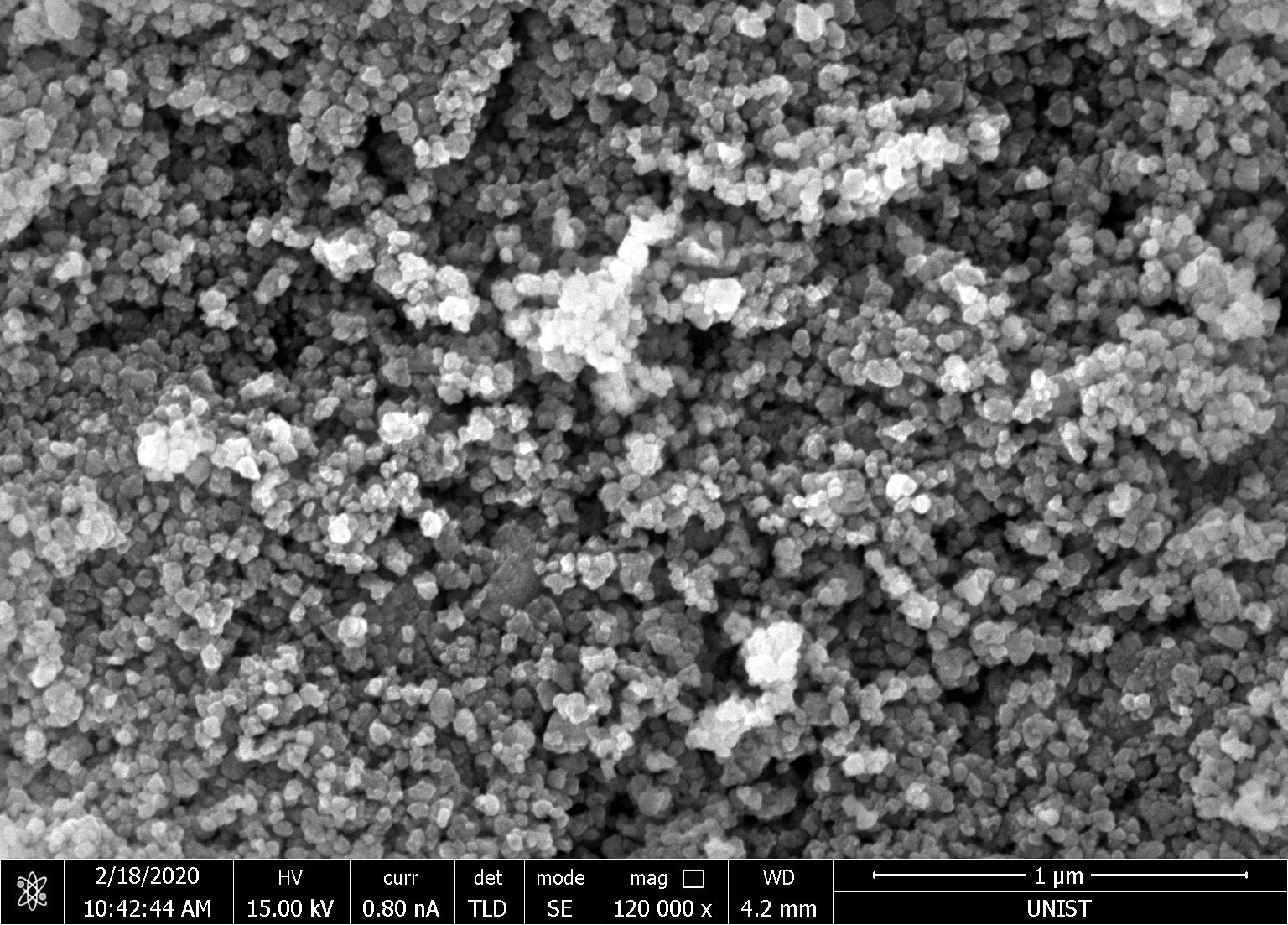

Supplement: Supplementary file 3 — Source Data [file 41467_2022_28947_MOESM3_ESM.zip › source data-1/Figure 1/Figure 1b.jpg]

| 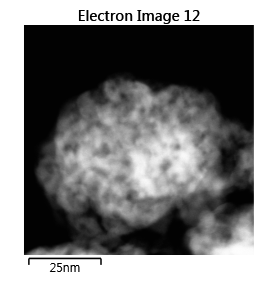 | 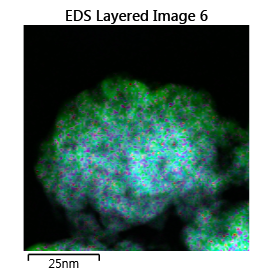 |
| --- | --- |


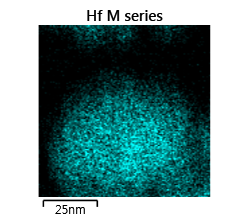

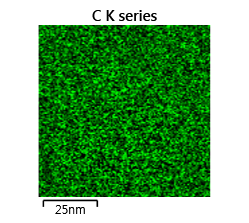

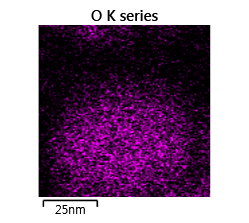

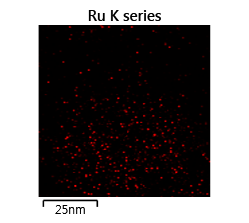

Supplement: Supplementary file 3 — Source Data [file 41467_2022_28947_MOESM3_ESM.zip › source data-1/Figure 1/Figure 1f-i.docx]

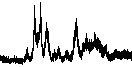

Supplement: Supplementary file 3 — Source Data [file 41467_2022_28947_MOESM3_ESM.zip › source data-1/Figure 1/Figure 1a/Vo-RuHfO2-OP.jip]

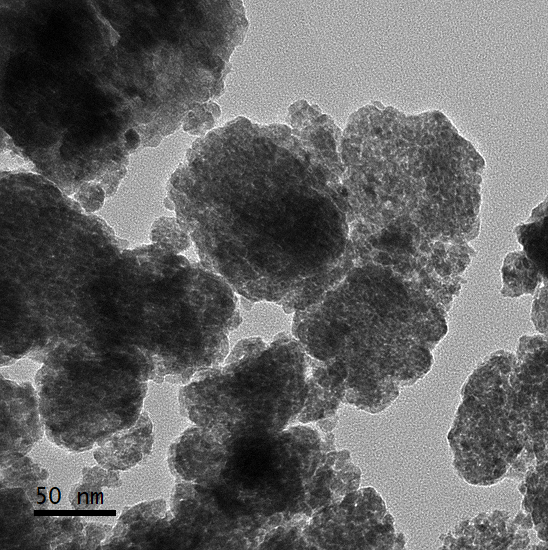

Supplement: Supplementary file 3 — Source Data [file 41467_2022_28947_MOESM3_ESM.zip › source data-1/Figure 1/Figure 1c.tif]

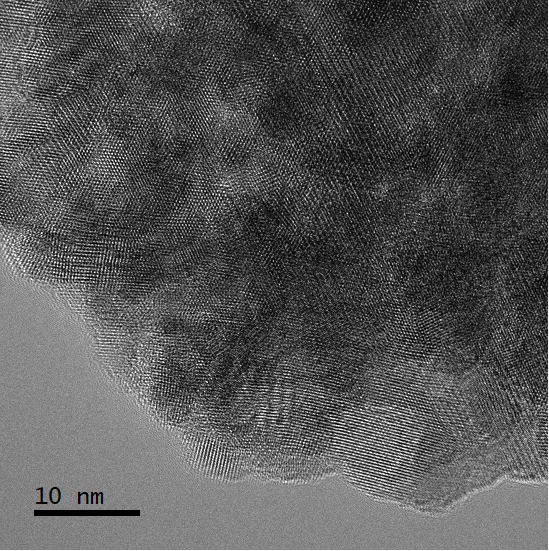

Supplement: Supplementary file 3 — Source Data [file 41467_2022_28947_MOESM3_ESM.zip › source data-1/Figure 1/Figure 1d-e.tif]

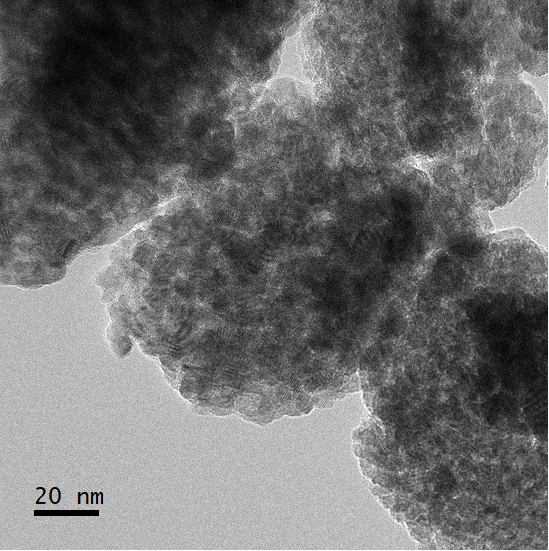

Supplement: Supplementary file 3 — Source Data [file 41467_2022_28947_MOESM3_ESM.zip › source data-1/Supplementary Figure 1/Supplementary Figure 1.tif]

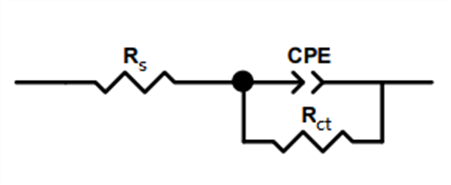

Supplement: Supplementary file 3 — Source Data [file 41467_2022_28947_MOESM3_ESM.zip › source data-1/Supplementary Figure 15/Equivalent circuit.tif]

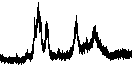

Supplement: Supplementary file 3 — Source Data [file 41467_2022_28947_MOESM3_ESM.zip › source data-1/Supplementary Figure 2/HfO2.jip]

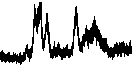

Supplement: Supplementary file 3 — Source Data [file 41467_2022_28947_MOESM3_ESM.zip › source data-1/Supplementary Figure 3/Vo-RuHf-O.jip]

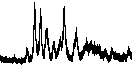

Supplement: Supplementary file 3 — Source Data [file 41467_2022_28947_MOESM3_ESM.zip › source data-1/Supplementary Figure 3/Vo-RuHf-P.jip]
