# Supplementary material for: The synergistic effect of Hf-O-Ru bonds and oxygen vacancies in Ru/HfO2 for enhanced hydrogen evolution
Source: Nat Commun. 2022 Mar 11;13:1270. doi: 10.1038/s41467-022-28947-9 (PMC8917135; doi:10.1038/s41467-022-28947-9)
Supplement: Supplementary file 3 — Source Data [file 41467_2022_28947_MOESM3_ESM.zip › source data-1/Supplementary Table 1/EXAFS_Fitting.docx]

Fitting results and parameters for ruthenium EXAFS (Oxygen = O, Ru or Hf = M) .

|  | Bond | Coordination number (CN) | Bond length  R (Å) | σ^2^ (Å) x 10^-3^ | E_0_ (eV) | R factor |
| --- | --- | --- | --- | --- | --- | --- |
| Ru foil | Ru-M | 12 | 2.662 (7) | 4.0 (6) | 2.5 (7) | 0.00169 |
| RuO_2_ | Ru-O | 6 | 1.980 (7) | 1.5 (6) | 1.1 (1) | 0.00099 |
| V_O_-RuHfO_2_-O | Ru-M | 5.0 (6) | 2.645 (9) | 8.8 (6) | 4.9 (6) | 0.00343 |
|  | Ru-O | 2.9 (2) | 2.023 (1) | 1.5 (9) | 2.8 (4) | 0.00066 |
| V_O_-RuHfO_2_-OP | Ru-M | 4.9 (5) | 2.653 (0) | 8.3 (3) | 4.2 (3) | 0.00379 |
|  | Ru-O | 3.4 (2) | 2.022 (8) | 1.0 (1) | 2.9 (1) | 0.00116 |
| V_O_-RuHfO_2_-P | Ru-M | 11.0 (1) | 2.674 (6) | 4.5 (1) | 6.9 (1) | 0.00376 |
|  | Ru-O | 0.48 (9) | 2.352 (8) | 7.1 (7) | 6.1 (2) | 0.00076 |
